# The development of chrome free chestnut and Tetrakis Hydroxymethyl Phosphonium Sulfate based Eco-benign combination tanning system

**DOI:** 10.1016/j.heliyon.2023.e23141

**Published:** 2023-12-06

**Authors:** Haftom Girmay, Ashagrie Mengistu, Berhanu Assefa, Mikiyas Abewaa, Getaneh Andualem, Bereket Yeheyis

**Affiliations:** a The Federal Democratic Republic of Ethiopia Manufacturing Industry Development Institute, P.O. Box 1180, Addis Ababa, Ethiopia; b School of Chemical and Biochemical-Engineering, Addis Ababa Institute of Technology, Addis Ababa University, P.O. Box 1176, Addis Ababa, Ethiopia; c Department of Chemical Engineering, College of Engineering and Technology, Wachemo University, Hossana, Ethiopia; d Manufacturing Industry Development Institute, Leather and Leather Products Industry Research and Development Center, P.O. Box 24692 code 1000, Addis Ababa, Ethiopia

**Keywords:** Chestnut, Combination tanning, THPS, Upper leather

## Abstract

The replacement of the conventional chromium tanning system with an eco-benign tanning systems has received great attention owing to stringent environmental regulations. In this specific study, a chrome-free combination tanning system based on chestnut and tetrakis (hydroxymethyl) phosphonium sulfate (THPS) was developed and presented as an effective alternative cleaner tanning technology. Processing of the tanning trials were carried out varying the percentages of chestnut as a tannage, followed by THPS as a re-tannage for process optimization. The leathers obtained were characterized for their thermal stability, grain surface properties using a scanning electron microscope, physical strength characteristics, comfort, and organoleptic properties. Finally, the environmental impact of the tanning systems was assessed through the comparative analysis of their spent liquors. The chestnut-THPS combination system tanned leathers using 20 % chestnut followed by 2 % THPS resulted in maximum shrinkage temperature of 95OC. The hydrothermal stability of the leathers tanned using this combination tanning system were found to be better than those tanned using chestnut and THPS tanning systems alone, respectively. The strength and comfort properties of the leathers produced using the developed combination tanning system were found to be on par with or better than those of conventionally tanned leathers, and the scanning electron microscopic study depicted that the grain surface of the leathers produced were observed to be free of surface deposition. The environmental impact assessment showed that the combination tanning system used resulted in a significant reduction in TS, TDS, TSS, and BOD in the wastewater. This research article has attempted and established the use of chestnut-THPS-based combination tanning systems as an effective, eco-friendly alternative tanning process technology.

## 1 Introduction

Nowadays, tannery industries are playing a significant role in the economies of nations through the production of finished and semi-processed leather products, which are important materials to make leather goods, garments, and footwear. In the course of producing these leather products, tanning is the core process used by tanners to change the purifiable hide or skin to non-purifiable intermediate products by stabilizing the triple helical structure of collagen matrix [[1], [2], [3]]. This chemical modification of the skin/hide collagen structure is supported by the functional groups on the side chains of the three polypeptide chains that form the spiral helical structure of the collagen protein [4,5]. In general, tanning uses functional groups on the side chains of polypeptides to chemically modify the collagen protein's structure with the more important effect of the forming multi-point cross-links between the tanning molecules and skin collagen fibers. These modifications enable the collagen to be turned into numerous products that are useful and necessary in the modern society [5]. Tanning agents are inorganic chemicals such as chromium, aluminum, and zirconium; organic materials such as vegetable tannins (i.e., mimosa, tara, chestnut, and valonea); phosphonium salts; and aldehydes that have the capability of reacting with the collagen fibers and alter the collagen structure, making it soft; strong; resistant to water, heat, and corrosion and chemically stable due to their molecular structure and cross-linking interaction made with the collagen structure [[5], [6], [7]].

Conventional chrome tannage is still the most commonly preferred and popular tanning system, and more than 90 % of the 2 billion m^2^ of leather produced worldwide is tanned using chromium [8]. This is due to the excellent tanning properties of chromium and the excellent qualities of chrome-tanned leathers, such as good affinity and compatibility with dyes, retanning materials, fat liquors, and finishing auxiliaries; high hydrothermal stability; resistance to external conditions of putrefaction; good strength characteristics and organoleptic properties and versatility [9]. As a transition element, chromium has 3D orbitals that induce the capability of taking extra electrons and forming coordination complexes that react with the ionized carboxyl group of the collagen via covalent bonding and undergo self-polymerization via hydroxyl bridges, forming a final stable -Cr-O-Cr bridge between the protein chains. Shortly, chromium in its Cr^3+^ form has the ability to carry out redox and substitution reactions as well as the ability to form coordinated aqua ligands to ionize into hydroxyl and polymerize into larger species in the PH range of 4–6 [6,10]. In addition, chromium can easily be obtained at a low cost. However, the limitations of inorganic tanning systems necessitate the development of organic tanning systems. Despite being the best mineral tanning agent, chromium has a number of negative images regarding its side effects on human health and the ecological environment. Cr (III) has the potential to oxidize to Cr (VI), or hexavalent chromium, in a moist environment, which has been proven to be carcinogenic, mutagenic, and highly toxic, causing allergic dermatitis as well as damage to the skin, mucous membranes, and respiratory tract [8,11]. Chromium (III) in leather products might be changed to Cr (VI) under some extreme conditions. The dumping of liquid and solid wastes containing residual chromium is becoming the main threat to human health and the ecological environment, bringing about difficulties for tanners regarding the compliance with the emerging environmental regulations [12]. Leather products have received a lot of attention as a source of chromium allergy and dermatitis since the 1990s [13,14]**.** After nickel and cobalt allergies, contact allergy to chrome is the third most common metal allergy, affecting 1–3% of the adult population [15,16]. Skin allergies, dryness, fissured skin, skin ulcers, and puffiness are all symptoms of prolonged skin exposure [17]. Dizziness, developmental issues, reproductive issues, discolorations, and tooth erosion are some of the other side effects of chromium [2,18]. In addition to these, the chromium tanning system tends to increase the chemical oxygen demand (COD), total dissolved solids (TDS), and sulfate content of spent chrome liquor [19].

Over the last 25 years, different tanning systems and technologies including titanium, silicon, and aluminum tannings, chromium exhaustion, recycling of basic chromium sulfate and partially replacing it with other organic tanning agents have been developed to avoid toxic chromium compounds [[20], [21], [22]]. These approaches, however, have their own set of drawbacks, such as organic salts on the final leather, inadequate chromium removal and the potential for human and animal health hazards due to lack of total replacement of chromium salts to elude adverse effects associated with chrome tanning [11]. Gradual increase in the environmental health and safety concerns is prompting tanners to look for and exercise an eco-benign alternative tanning system that are cleaner, safer, and more viable. An eco-friendly tanning technologies such as the application of green tanning agents free of chromium and other metal tanning agents have drawing due attention with great improvement in environmental awareness, and there has been a growing requirement for chrome-free leather products such as upholstery leather, garment leather, and automotive leathers [23,24].

Recently, a considerable number of scholars have tried to replace chromium and other metal based tanning systems with organic vegetable tanning agents, phosphonium salts and aldehyde combination tanning systems. Where, the most common ones are, dialdehyde alginic acid and THPS combination tanning system developed by Ref. [25], henna-glutar aldehyde combination tanning system by Ref. [26], glutaraldehyde and THPS combination tanning system by^26^ and garad and glutaraldehyde combination tanning system by Ref. [27]. However, the use such combination tanning systems end up with free aldehydes on the surface of the final leather and their presence in leather even at the level of 50 ppm is becoming a huge cause of concern for leather manufacturers [28]. On the other hand, the use of tara vegetable tannin as a combination tanning agent with THPS was used as a scavenger of free formaldehyde present in THPS tanned leather, However, the tanned leather product was observed to be infected with fungus [29]. Therefore, the is a serious need to search for an organic tanning agent that can be used with THPS in order to develop an eco-benign combination tanning system for the production of green leather free of aldehydes.

**Fig. 1:**
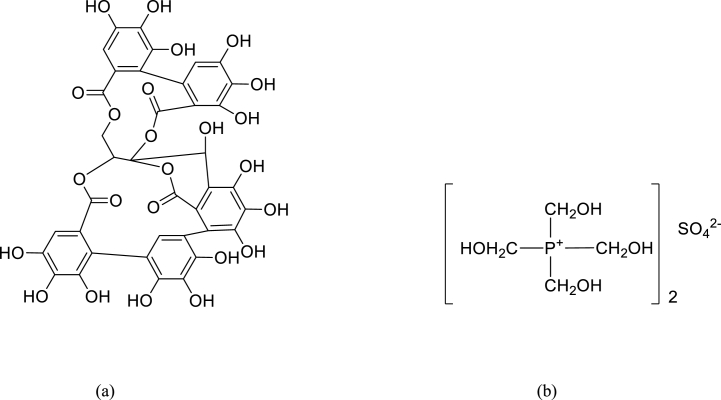
(a) Representative structural diagram of chestnut tannin (b) Structural formula for THPS.

**Fig. 2:**
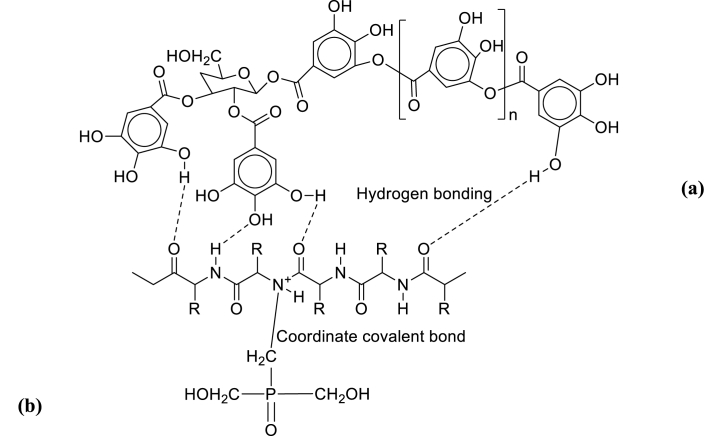
Schematic diagram showing interaction of (a) chestnut and (b) THPS with amino acid group of collagen fiber.

In this present study, chestnut and tetrakis hydroxymethyl phosphonium sulfate (THPS were chosen for the chrome-free combination tannage experiment. Chestnut is a glucosidic tannin that is extracted from chestnut wood (Castanea sativa) with a molecular formula of C_15_H_16_O_9_ and structural formula as shown in Fig. 1(a). It is a pyrogallol tannin that belongs to a category of easily hydrolyzed glucosidic tannins [30,31]. Chestnut extracts have a high concentration of acid groups and natural organic acids, which contribute to their astringency and ability to remove larger scales from the hide. These properties allow chestnut extract to produce a leather that is compact, firm, versatile or flexible, waterproof, and has a high affinity for pelt, allowing for a high tannin fixation [21,32]. It has been established that the interaction between vegetable tannin and collagen is primarily based on the cooperative action of hydrogen bonds and hydrophobic bond as well as the physical adsorption of colloidal tannin; the former is primarily responsible for enhancing collagen's hydrothermal stability [33] as shown in Fig. 2.

Additionally, THPS tanning agent and vegetable tannin undergo a further reaction with one another that results in polymerization. This polymerization is the reason for the high hydrothermal stability, which permits the collagen fibers to build a multiple-bond tanned matrix inside of them [33].

THPS is a colorless organic liquid that has the molecular formula of C_8_H_24_O_12_P_2_S and structural formula as shown in Fig. 1(b). It is used produce leather with an excellent light fastness, pelt with a very good penetration, light shades, a good tanning effect, and a white appearance, allowing for the production of leathers with a variety of colors, excellent aesthetic properties, and better physical properties^30^. The use of tetrakis hydroxymethyl phosphonium sulfate (THPS) as an alternative tanning system to chromium tanning systems has been investigated, and it was observed to enhance the shrinkage temperature of lambskins^19,34^. In addition, combination tanning of THPS with iron was found to alleviate the problems associated with iron tanning, such as darkening of the iron-tanned leathers and loss of their strength characteristics up on ageing [19]. Furthermore, the use of THPS in a combination tanning system is beneficial due to its low toxicity, low bioaccumulation due to its rapid biodegradability, and reduced risk to human health and the ecological environment [19]. Its interaction with amino acid group of collagen fiber through its hydroxymethyl group to form coordinate covalent bond was as depicted in Fig. 2.

The research work considers the development of a chrome-free, eco-friendly combination tanning system based on chestnut and THPS being applied to produce sheep upper leather, where an intensive study on the optimization of dosage of the two tanning materials, the physico-chemical characterization of both experimental and control leathers, and the environmental impact analysis of the spent liquor from the tanning system were attempted.

## 2 Materials and methods

### 2.1 Raw materials and chemicals

Wet salted sheepskins of Indian origin were procured from a slaughterhouse in Perambur, Chennai, and analytical-grade chemicals and reagents for laboratory experiments such as sodium hydroxide pellet (NaOH), 99.0 %; hydrochloric acid, 37 % w/w (HCl); sulfuric acid (H_2_SO_4_), 98 %; nitric acid (HNO_3_), 65 %; perchloric acid, 70 %; sodium chloride (NaCl), 99.5 %; boric acid, 99.5 %; basic chrome sulfate (33 % basicity); and tanning chemicals were obtained from the CLRI tannery laboratory. Laboratory equipment and instruments mainly used in this study were a round bottom flask, a water bath, a digital electronic balance, a digital pH meter, an analytical weighting balance, a hot air oven, testing equipment such as a scanning electron microscope (SEM), and machines to study the mechanical strength characteristics of the crust leather. The leather processing chemicals were all commercial grade, while the reagents and chemicals employed for laboratory analysis were analytical grade. Chestnut and tetrakis hydroxymethyl phosphonium sulfate (THPS) were obtained from CLRI model tanneries and laboratories, respectively.

### 2.2 Methods

#### 2.2.1 Preparation of the pickled pelt

Before the application of the tanning agents, the 33 pieces of wet salted sheepskins were conventionally processed until pickled, based on the formulation shown in Table 1. The pickling process prepares the pelt for the chrome tannage in which the pH of the pickle solution is usually adjusted to pH value of 2.8–3.2 depending on the tannage and the type of raw material. Pickling is used to slows down the astringency of chrome tanning process which helps to prevent the tanning agents from binding to the external pelt layers, as this would stop their deeper penetration. During pickling process, acid contained in the pickle solution reacts with the basic functional groups of collagen side chains and imparts to it a lower required pH of about 2.8–3.2 at which better penetration of chrome is obtained. In addition, pickling is helps to get rid of the unwanted protein in the animal skin, which helps the tanning chemical to better adhere to the skin [34,35]. A total of eleven experimental runs and one control run were performed, of which nine were of combination tanning systems examined after the pH of the pickle float was adjusted to 2.8–3.2, and two were done with chestnut and THPS alone. Three pieces of skin were taken per single run, then divided along the back bone in order to have materials of the same property for both the experimental and control runs. The control tanning was carried out concurrently using basic chromium sulfate (33 % basicity). The pre-treated pelts were used as raw material for both experimental and control tanning trials. The conventional tanning parameters were applied both for the experimental and control processes. Finally, tanning spent liquors from both control and experimental processes were collected to study the environmental impact assessment by testing for TSS, TDS, BOD5, and COD. The butt portion of the tanned leather samples was taken for a shrinkage test. Finally, the samples were further processed until the crust leather for the sheep's upper was taken for physical strength characterization.

**Table 1:** Formulation for conventional process to prepare pickled pelt.

Process	Quantity (%)	Chemicals	Duration (min)	Remarks
Soaking	300	water	For 6hrs changing the water twice	Wash/drain
Liming using paste	10	Water		The paste was prepared at Baume meter reading of 10–12 and was applied on the grain side of the skins and piled for 6hrs and then unhaired.
	8	Lime		
	2	Sodium Sulfide		
Deliming	80	Water		Check deliming completion using phenolphthalein
	1	Ammonium sulfate		
	0.5	Sodium bisulfate	Run 60 min	
Adjustment of PH	1	Water		Check PH = 4.8 Drain 70 % of liquor
	0.5	Acetic acid	3 × 10 + 15'min	
Bating	0.4	Bating agent (Oropon ON_2_)	50 min	
Degreasing	3	Degreasing agent	45 min	
Pickling	30	Water (liquor)		Check penetration And record PH
	10	Chestnut	30 min	
	10	Chestnut	30 min	
	2	THPS	60 min	
**Adjustment of PH**	80	Water		Check the PH to be 2.8–3.2 and leave for a while for equilibration
	8	Sodium chloride		
	1.25	Sulfuric acid	10min	
	15	Water for dilution	2*15+2hrs	

#### 2.2.2 Optimization of Chestnut-THPS combination tannage

Thirty pieces of the prepared delimed pelt were taken for the nine experimental runs of optimizing the percentage dosage of the combination tanning system and the other three runs, where three pieces of pickled pelt were used for each single run, being divided along the back bone. The other half of the delimed pelts were used as a control leather sample after being further processed into pickled pelts. The delimed pelts were tanned with three different percentages of chestnut, viz., 10, 15, and 20 %, where the PH was corrected to 5 using 0.5 % diluted acetic acid, and then the sample was drummed for 60 min. After the completion of the chestnut tanning processes, each experimental sample was further tanned using the following three different percentage dosages of THPS (1, 1.5, and 2 %) based on the designed experiment, as shown in Table 2, then further drummed for another hour. The rest of the half sides of the pelts were further processed until the pickled pelt was conventionally tanned using chromium as a tanning agent. Basification was carried out using 0.5% sodium formate and 0.75% sodium bicarbonate at a dilution rate of 1:10, applied with three feeds at a 10-min time interval, i.e., drumming for a total of 30 min. After running the drum for 2 h, the PH was discovered to be between 3.8 and 4.0. The tanned skins were cleaned with 20 % water. Samples of spent liquor were taken from each trial for environmental impact assessment, and the tanned leathers were then piled for 24 h.

**Table 2:** The tanning trial runs and their percentage offers of experimental and control processes.

Trial number	Percentage offers of chestnut (%)	Percentage offers of THPS (%)
1	15	1
2	10	1
3	20	1.5
4	20	1
5	10	2
6	15	2
7	10	1.5
8	20	2
9	15	1.5
10	20	2.5
Controls	Percentage offers of controls (%)	
Chestnut alone	20	
THPS alone	2	
Chromium	6	

After 24 h of piling, the thermal stability of the wet-tanned leathers was measured using theis shrinkage tester, where each value was reported as an average of three separate measurements with their standard deviation as shown in Table 3.

**Table 3:** Formulation of Chestnut-THPS tanning system for the experimental runs.

Process	%	Chemicals	Duration (min)	Controls
Deliming	80	Water		Check completion using phenolphthalein
	1	Ammonium sulfate		
	0.5	Sodium bisulfate	Run 60 min	
Adjustment of PH	1	Water		Check PH = 4.8 Drain 70 % of liquor
	0.5	Acetic acid	3 × 10 + 15'min	
Bating	0.4	Bating agent (Oropon ON_2_)	50 min	
Degreasing	3	Degreasing agent	45 min	
Tanning	30	Water (liquor)		Check penetration And record PH
	10	Chestnut	30 min	
	10	Chestnut	30 min	
	2	THPS	60 min	
Adjustment of PH	0.5	Sodium formate		Check the PH to be 3.8–4
	0.75	Sodium bicarbonate	3 × 10 min	
	15	Water	120 min	
**Drain the bath and pile overnight. Next day sammed and shaved to 1.1 to 1.2 mm.**				

#### 2.2.3 Conventional tanning process

The left-half sides of the thirty delimed pelts and the other three experimental samples for THPS alone were further processed till pickled pelt, tanned chromium, and THPS, respectively, as shown in Table 4. Three half sides of the delimed pelt were also taken for chestnut tanning. The recipe for tanning purposes is depicted in Table 4.

**Table 4:** Formulation of Chestnut alone, THPS alone and conventional tanning systems for upper leathers.

Process	%	Chemicals	Duration (min)	Controls
Chestnut tanning	30	Water (liquor)		Check penetration And record PH
	10	Chestnut	30 min	
	10	Chestnut	30 min	
	0.5	Formic acid	3 × 10 min	
	15	Water	120 min	
THPS tanning	50	Pickle liquor		Check PH to be 3.8–4.
	2	THPS	50 min	
	1	Sodium formate	15 min	
	1.5	Sodium bicarbonate	3 × 10 min	
	15	Water	120 min	
Chrome tanning	50	Pickle liquor		Check penetration and PH to be 3.8–4.
	6	Chromium	60 min	
	1	Sodium formate	15 min	
	1.5	Sodium bicarbonate	3 × 10 min	
	15	Water	120 min	
**Drain the bath and pile overnight. Next day sammed and shaved to 1.1 to 1.2 mm.**				

#### 2.2.4 Wet-finishing operations for control and experimental leathers

The wet-finishing process of sheep upper leather involves a number of operations, including rehydration, neutralization, re-tanning, dyeing, fat-liquoring, and fixing as shown in Table 5. First, the tanned leathers were subjected to through feed samming machine to remove the internal water, then shaved to a uniform thickness. In this operation, a common wet-finishing operation was carried out for all the experimental (Chestnut-THPS), chestnut (alone), and chrome-tanned control leathers.

**Table 5:** Wet-finishing recipe for both experimental and control upper leathers.

Process	%	Chemicals	Duration(min)	Controls
Neutralization	150	Water		Check PH to be 5–5.2
	1	Sodium formate		
	0.75	Sodium bicarbonate	3 × 10 + 30 min	
Re-tanning	100	Water		
	2	Acrylic syntan (Relugan RE)	20 min	
	6	Melamine resin syntan (Basyntan FB6)		
	5	Phenolic syntan (Basyntan DI)	60min	
Fat-liquoring	100	Water		
	5	Synthetic fat-liquor		
	4	Semi synthetic fat-liquor		
	2	Lecithin fat-liquor	45 min	
Dyeing	4	Acid black dye	50 min	Check penetration
Fixing	1	Formic acid	3 × 10 + 30 min	Check fixation
**Pile overnight and next day go to: Setting, hook to dry, stacking, trimming and buffing.**				

#### 2.2.5 Analysis methods

##### 2.2.5.1 Measurement of hydrothermal stability

The resistance of the material to wet heat is measured by hydrothermal stability. The temperature at which control and experimental leathers shrank was determined using a shrinkage tester. From the sample position, a 2 × 0.5 cm^2^ piece of tanned leather was cut and clamped between the clamp's jaws before being soaked in a 3:1 glycerol: water solution. The solution was constantly mixed using a mechanical stirrer linked to the shrinkage tester. The temperature of the solution was steadily increased, and the temperature at which the sample shrank was recorded as the shrinkage temperature of the leathers.

##### 2.2.5.2 Scanning electron microscopic analysis

Control and experimental tanned crust leathers were analyzed using a scanning electron microscope to examine the influence of combination tannage on the fiber structure and to understand the surface and cross-section fiber compaction. The official sampling location was used to cut samples from experimental and control crust leathers. A Quanta 200 series scanning electron microscope was used for the investigation. A scanning electron microscope (SEM) in low vacuum with a 20 kV accelerating voltage and various magnification settings was used to produce grain surface and cross-section micrographs.

##### 2.2.5.3 Reflectance measurements

The reflectance of the experimental and control crust leather was measured based on the principle of measuring and evaluation of the fraction of the amount of light reflected from the surface of an opaque specimen at various visible spectrum wavelengths with respect to an identically illuminated white standard [36]. In this investigation, the Techkon Spectro drive instrument was used to measure the reflectance of the color of the control and experimental leathers, where the L*, a*, b*, c*, and hue* values are the parameters used to assess and evaluate the color of leather samples in the CIELAB color space. L* represents whiteness, a* represents the red and green axes, where a*< 0 means green and a* >0 means red, b* represents the yellow and blue axes, where b*< 0 means blue and b*> 0 means yellow, and c* represents the chromacity of the color, which means the intensity of the color. The values reported are the average of the three values. Equations (1), (2)), respectively, were used to calculate the overall color difference (ΔE) and hue difference (Δh) [36].

(1) $$\Delta E=\sqrt{{L^{*}}^{2}+{a^{*}}^{2}+{b^{*}}^{2}}$$

(2) $$\Delta H=\sqrt{{\Delta E}^{2}-{{\Delta L}^{*}}^{2}-{{\Delta b}^{*}}^{2}}$$

Where, ΔE, is the overall color difference, $\Delta L^{*}$, is the difference in lightness, Δa and Δb, are difference in a and b values and ΔC is the chromaticity difference. Parameters like ΔL, Δa, Δb,and ΔC were determined by subtracting the corresponding values of leather produced from those of control leather.

##### 2.2.5.4 Analysis of spent liquors from tanning trials

The wasted tannin liquor from the control and experimental tanning operations was collected and filtered, then COD, BOD5, TDS, TSS, and TS were measured as per the standard procedures [36,37,38]. The amount of effluent (lit) per tonne of raw skins processed was multiplied by the concentration (mg/l) to calculate emission loads. The average of three experiments was reported based on their standard deviations.

##### 2.2.5.5 Physical characterization

IULTCS procedures were used to obtain samples for various physical tests from experimental and control crust leathers [[39], [40], [41]]. A vital stage in the physical testing of leather is conditioning a leather sample or a leather cut test specimen. The amount of moisture in the leather matrix varies depending on how humid the working environment is. Another well-known parameter that determines matter's qualities is temperature. For two days, specimens were conditioned at 20 ± 2 °C and 65 ± 2 % relative humidity. Tensile strength, percentage elongation at break, grain crack strength, and tear strength were all determined using established methods [42,43]. Each value was calculated based on an average of four samples (two along the backbone and two across the backbone).

##### 2.2.5.6 Determination of comfort properties

It is known that hygienic properties are the most important parameter for any form of leather because they only distinguish or separate natural leather from synthetics. Hence, it is essential to examine the comfort properties of the control and experimental leathers. The most frequently used tests to measure the comfort properties of shoe upper leather are water vapor permeability (WVP), water vapor absorption (WVA), and water penetration resistance (WPR). The comfort properties were analyzed as per the standard procedures [[44], [45], [46]].

##### 2.2.5.7 Organoleptic properties of crust leathers

Hand and visual evaluation were used to assess the softness, fullness, grain smoothness, grain tightness (break), general appearance, and dye uniformity of experimental and control crust leathers. For each functional attribute, three professional tanners graded the leathers on a scale of 0–10 points, with higher numbers indicating greater quality.

##### 2.2.5.8 Chemical characterization

For control and experimental leathers, chemical testing methods such as percentage of moisture content, percentage of oils and fats content, percentage of hide substance, and percentage of total ash content were carried out as per the standard procedures [47].

## 3 Results and discussions

### 3.1 Tanning optimization using Chestnut-THPS combination tannage

Thies shrinkage tester was used to determine the hydrothermal stability of samples leathers tanned using combination and conventional chromium tanning systems. Combination tanning system was optimized using chestnut-THPS with different percentage offers of chestnut (10, 15, and 20 %) and THPS (1, 1.5 and 2 %), respectively. Combination tanning using chestnut and THPS was found to be successful after many trials. Eleven trials were conducted based the previously done works to optimize the combination tanning system using shrinkage temperature of the tanned leather, environmental impact assessment using the spent liquors both from the experimental and control processes and organoleptic properties of the crust leather after post-tanning as response parameters, with the process that gave the highest shrinkage temperature being chosen as the optimal process recipe^20^. The shrinkage temperature of the leathers tanned using combination tanning system considering different dosages of the tanning agents and the control leathers at constant percentage offer under conventional chromium tanning systems is shown in Table 6 in the form of a mean ± standard deviation.

**Table 6:** Shrinkage temperature of Chestnut-THPS experimental and control tanned leathers.

Trial number	Offers of Chestnut (%)	Offers of THPS (%)	Mean shrinkage temperature (^o^c)
1	15	1	88 ±1.2
2	10	1	86 ±1.6
3	20	1.5	92 ±1.1
4	20	1	90 ±1.6
5	10	2	87 ±1.5
6	15	2	91 ±1.2
7	10	1.5	87 ±1.7
8	20	2	95 ±1.5
9	15	1.5	89 ±2
10	20	2.5	96 ±1
Controls	Percentage offers (%)		Shrinkage temperature (^o^c)
Chestnut (alone)	20		82 ±1.4
THPS (alone)	2		84 ±1.4
BCS	6		105 ±1.5

As it was observed from the tabulated result, leathers tanned using chestnut-THPS combination tanning system using 20 % chestnut followed by 2 % THPS analyzed to have maximum hydrothermal stability of 95 °C. However, the recorded shrinkage temperature while conducting the tanning process using chestnut and THPS separately was observed to be 82 °C and 84 °C, respectively. In general, the optimization results of the chestnut-THPS combination tanning system end up with tanned leather with a shrinkage temperature of 11 °C–13 °C higher than those tanned with chestnut and THPS alone. It was also observed that a percentage increase in the offer of one of the tanning agents while keeping the other constant produces thermally more stable leather with a significant increase in shrinkage temperature. However, a further increase in dosage of THPS beyond 2 % resulted in coarser-grain leather with an insignificant increase in shrinkage temperature [48]. In general, the 20 % offer of chestnut followed by 2 % THPS was observed to be the optimum percentages of the developed combination tanning system with a view to increasing the hydrothermal stability of tanned leather together with the smoothness of the grain leather. Similar investigations were conducted by scholar using THPS combination tanning system with henna [36], silica-aluminum [49], tara [29], Fe [48], aluminum [50] and minimized amount of chromium [51] and the shrinkage temperatures of the tanned leathers were obtained to be 96, 86, 88, 95, 88 and 110 °C, respectively. However, such combination tanning system were not Eco-benign and tanning agents were observed in the tannery effluent and on the surface of the leather product. Tara acts as scavenging agent for aldehydes on the surface of leather, however, the produced leather was observed to be susceptible to fungus.

### 3.2 Scanning electron microscopic studies on crust leathers

**Fig. 3:**
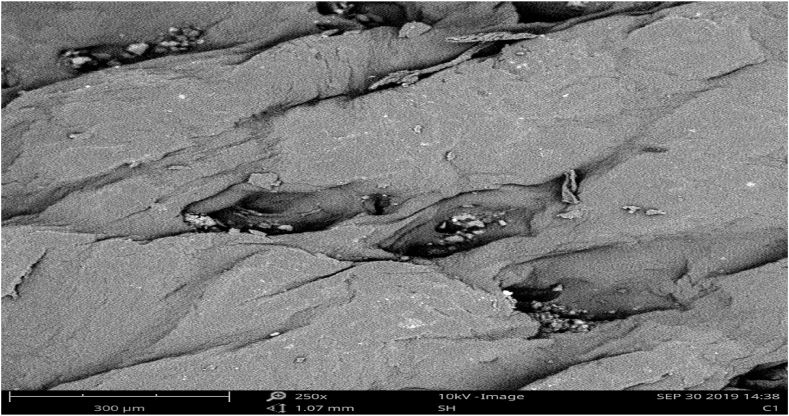
SEM micrographs of the grain surface (x250) of upper crust leather control.

**Fig. 4:**
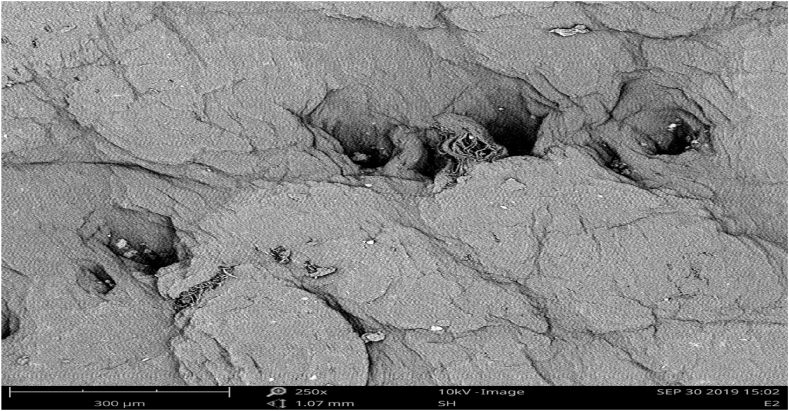
SEM micrographs of the grain surface (x250) crust leather for experimental.

**Fig. 5:**
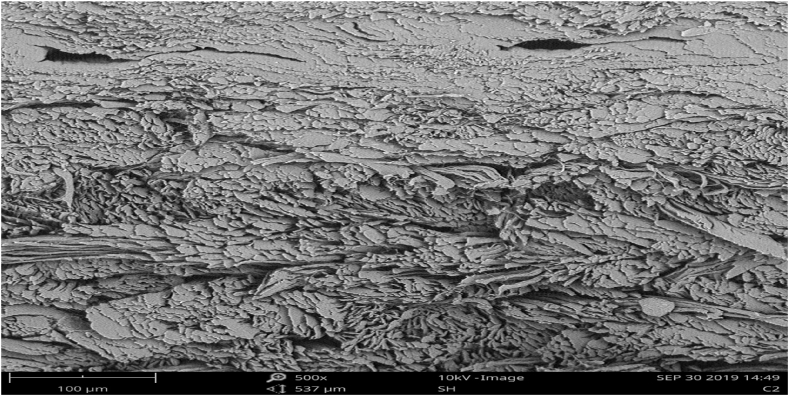
SEM micrographs of the cross-section (x500) of crust leather for control.

**Fig. 6:**
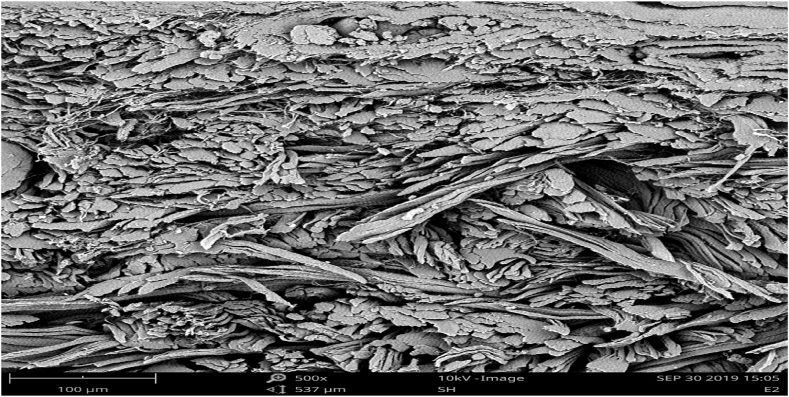
SEM micrographs of the cross-section (x500) of crust leather for experimental.

The surface pattern and cross-sectional structure for both the experimental and control samples are as shown in Fig. 3, Fig. 4, Fig. 5, Fig. 6. The scanning electron microscope analysis provides information regarding the fiber compactness and the grain surface patterns of the leather samples, which vary due to the types of chemicals applied at different stages of leather processing. Different magnifications, like ×250 and x500, were used to enhance the visibility. Scanning electron microscopic analysis (SEM) was carried out on the crust leather samples tanned using chestnut-THPS and the conventional chrome tanning systems to investigate the effect of the two tanning systems on the fiber structure and to understand the grain surface pattern and cross-sectional fiber compactness. The SEM analysis show the scanning electron microscopic images of the cross-section and grain surface of the crust leathers produced using both the combination and conventional tanning systems with magnifications of 500× and 250X, respectively. The presence of THPS in the combination tanning system provided that the final crust leather had good structural stability and smoothness due to its fiber coating property in the leather matrix [52,53]. Scanning electron microscopic (SEM) analysis of the grain surface of the combination tanned crust leather at 250× magnification proved the absence of grain surface deposition of tannins. Manual evaluation of the crust leather using hand felling and visual inspection proved the leather to have good fiber compaction and grain tightness properties. In general, the filling nature of chestnut also seen to give the crust leather an admirable fullness [54].

As it clearly depicted in the Scanning Electron microscopic micrograph shown in Fig. 3, Fig. 4, the grain surfaces of leathers tanned using both the conventional and combination tanning system were comparably clean, with less foreign particles on the grain surface of those tanned using combination tanning system. It was also displayed in Fig. 5, Fig. 6 of the X500 magnification of scanning electron micrograph that the cross section of both crust leathers was seen to have comparable fiber structure with clear fibers were observed in the experimental crust leathers.

### 3.3 Evaluation of reflectance measurements of the upper crust leathers

The reflectance measurements were carried out for the chestnut-THPS, conventional chromium, and chestnut tanned crust leathers to study the effect of surface color on the control and experimental leathers, where L*, a*, b*, and hue* were taken to be the values of parameters used to assess and evaluate the intensity of color shade of leather samples, as shown in Table 7. L* represents whiteness on a scale ranging from 0 to 100, in which 100 on the scale represents pure white [48]. The maximum L* values for chestnut-THPS combination tanned crust leather (experimental), chestnut alone tanned crust leather (experimental), THPS alone tanned crust leather (experimental), and conventional chromium tanned crust leather (control) were evaluated to be 34.559, 32.793, 67.164, and 37.363, respectively. These data describes that the color hue of the crust leather tanned using THPS alone is lighter in shade compared with the rest processed with the other three tanning systems where, similar results were obtained in investigations in which THPS was given priority of application during the combination tanning system showing that the leather's lightness [48,36]. On the other hand, the a* values for the crust leather obtained from the leather produced using combination tanning system of chestnut-THPS, chestnut alone, and THPS alone were analyzed to be 0.178, 0.144, and 0.101, respectively. where a* represents the red and green axes, in which a*< 0 means green and a* >0 means red, which indicates that all the colors obtained have a red shade similar to the one reported by Refs. [48,36] when henna was given priority of application during the experimental combination tanning system and it becomes greener when THPS was applied first. However, the conventionally tanned control crust leather has a green shade with a* = -0.302. The b* values for leathers tanned using chestnut-THPS tanning system, crust leathers tanned using chestnut alone, and conventionally tanned control crust leathers were all analyzed to be greater than zero, indicating that as b* denotes the yellow and blue axes, in which b* <0 shows blueness of the crust leather and b*> 0 describes the crust leather to have yellow shade. In this specific study, the result demonstrated by b* >0 indicates that all the crust leathers produced have a yellow shade which contradicts the one reported by Ref. [36] to be blue when henna was applied first in the combination tanning system with THPS. The intensity of the color, which is called chromacity, is represented by c*, where in this specific study the value of c* for all the crust leathers obtained using the experimental and control tanning systems has a color shade with less depth and intensity, making it appropriate for pastel hues, as shown in Table 7. In general, leather tanned using THPS alone found to be lighter, less red, less yellow, stronger chromacity showing an increase in hue value. On the other hand, chestnut-THPS combination system tanned leather was analyzed to be less light compared to the control leather, redder compared to the other experimental leathers, yellower, stronger chromcity showing a decrease in hue value.

**Table 7:** Reflectance measurement results for experimental and control crust leathers.

Leather samples		Parameters				
		L	a*	b*	c*	Hue
Chestnut-THPS		34.559	0.178	2.480	2.490	85.977
Chestnut (alone)		32.793	0.144	2.009	2.014	84.905
THPS (alone)		67.164	0.101	0.841	0.651	94.103
Chrome (control)		37.363	−0.302	1.042	1.085	86.851
Sample	ΔL	Δa	Δb	Δc	Δh	ΔE
Chestnut-THPS	−2.804	0.48	1.438	1.405	−0.874	3.188
THPS (alone)	29.801	0.403	−0.201	−0.434	7.252	29.804
Chestnut (alone)	−4.570	0.446	0.967	0.929	−1.946	4.692

### 3.4 Environmental characteristics of spent tan liquor

The analysis of spent liquor parameters such as chemical oxygen demand (COD), biological oxygen demand (BOD), total solids (TS), total suspended solids (TSS), and total dissolved solids (TDS) were conducted in a triplicate both for spent liquors obtained from drums used for combination and conventional chrome tanning system as shown in Table 8.

**Table 8:** Environmental characteristics of spent liquor from tanning processes.

Parameters (mg/l)	Leather samples tanned with			
	Chestnut-THPS (E)	Chestnut (alone)	THPS (alone)	Chrome (control)
TS	10540 ± 16	12092 ± 19	12239 ± 16	14731 ± 21
TDS	8014 ± 14	11276 ± 18	11402 ± 10	16426 ± 12
TSS	4912 ± 13	9017 ± 16	5961 ± 8	6431 ± 18
BOD	1485 ± 21	6531 ± 12	2125 ± 14	1809 ± 10
COD	3241 ± 10	17310 ± 13	3873 ± 11	2750 ± 16

As it was observed from the tabulated data analysis, the values of total solids (TS), total dissolved solids (TDS), total suspended solids (TSS), and biological oxygen demand (BOD) of spent liquor obtained from the chestnut-THPS combination tanning system are lower compared with those obtained from the conventional chrome tanning system. However, the value of COD is slightly higher, which might be due to the fact that vegetable tannins (chestnut) were reported to increase the COD level of spent liquor [[55], [56], [57]] which was observed in the experimental data when chestnut and THPS were used as a tanning agent alone. On the other hand, when compared with spent liquor from the drum in which chestnut and THPS were used for tanning separately, the values of COD, BOD, TS, TDS, and TSS were observed to dramatically reduce in the chestnut-THPS combination tanning system. This could be due to the enhancement of chestnut's exhaustion during combination tanning [36,54,56].

### 3.5 Physical strength characteristics of crust leathers

The physical strength characteristics of sheep upper crust leather, such as tear strength, load at grain crack, distension at grain crack, tensile strength, and percentage of elongation at break for both the experimental and control sheep upper crust leathers, were analyzed in triplicate using standard procedures [48] and the result is presented in Table 9 in the form of a mean and standard deviation. Tensile strength for experimental and control samples was tested using a universal tensile tester both along and across the backbone of the experimental and control crust leather samples [58]. The ultimate strength of leather is due to all three layers of grain, corium, and flesh and is the reason for the leather to withstand various mechanical operations such as shaving at the wet blue stage, staking during the crusting, and buffing of the intermediate leather product. determining the tensile strength of leather is a mandatory task in the leather sector [59,60]. Testing tear strength is an important task to estimate the durability of the leather to withstand tearing stresses encountered during the manufacturing of shoes, garments, gloves, and upholstered products. So, it is a more preferred type of test by customers than tensile strength [43,61].

**Table 9:** Physical strength characteristics of experimental and control crust leathers.

Parameters	Leather samples			
	Chestnut-THPS (E)	Chestnut (alone)	THPS (alone)	Chrome (control)
Tear strength (N/mm)	54.1 ± 2	53.5 ± 3	46 ± 3	47.3 ± 2
Load at grain crack (N)	456.2 ± 5	398.7 ± 5	387 ± 1	408.4 ± 3
Distension at grain crack (mm)	12 ± 0.5	10.6 ± 1.5	10 ± 0.5	11 ± 1
Tensile strength (N/mm^2^)	26.9 ± 1	30.7 ± 2	27 ± 2	28 ± 1.5
Elongation at break (%)	66.0 ± 2	62.8 ± 2	62 ± 1	64 ± 1

The tabulated data shows that the strength characteristics of the Chestnut-THPS tanned crust leathers, such as tear strength, load at grain crack, distension at grain crack, and percent elongation at break, were found to be higher than those tanned with Chestnut and THPS separately and the ones conventionally tanned using chromium tanning systems and reported in a similar manner by Ref. [36]. This could be due to a higher level of crosslinking between the skin matrix fiber structure and the tannins utilized in the combination tanning system [62,63]. whereas the tensile strength of chestnut-THPS tanned (E) crust leathers was found to be marginally lower than those tanned using conventional chrome tanning systems, chestnut (alone) and THPS (alone). Compared with the conventional chrome-tanned upper leathers, the chestnut-tanned leathers have superior tensile and tear strength. The strength parameters such as tear strength and grain crack strength are very important, particularly towards the durability of upper leather [42,43,64]. Thus, due to this, the optimized combination tannage (E) is the most preferable tanning system for the industrial production of upper leather.

### 3.6 Comfort properties of crust leathers

Water vapor permeability (WVP), water vapor absorption (WVA), and water penetration resistance (WPR) are the most often used tests to determine the comfort qualities of shoe upper leather (WPR). Standard protocols were used to analyze the comfort qualities. Table 10 shows the results of testing the hygienic properties of experimental and control upper crust leathers.

**Table 10:** Hygiene properties of experimental and control crust leathers.

Parameters	Leather samples			
	Chestnut-THPS	Chestnut (alone)	THPS (alone)	Chrome (control)
WVP (mg/cm^2^.h)	7.8	5.1	4.1	6.02
WPR (min)	0.9	0.8	0.7	1
%WVA (60 min)	58.9	48.7	46.5	51.2

The above hygienic features are known to be the most significant criterion for any type of leather because they are the sole way to distinguish natural leather from synthetics. The test results revealed that the leathers tanned using the chestnut-THPS (E) combination tanning system have higher values of water vapor permeability and percentage of water absorption than those tanned under the conventional chrome tanning system (C) and those tanned with chestnut and THPS separately, where the chestnut-tanned leathers show the lowest value of water vapor permeability. This result can be clarified by the higher density of the chestnut tannin particles, which cause lower vapor permeability. On the other hand, the value of water penetration resistance for both chestnut-THPS and chestnut-tanned leathers was found to be slightly lower than those tanned using conventional chrome tanning systems.

### 3.7 Assessment and hand evaluation of bulk properties of crust leathers

**Fig. 7:**
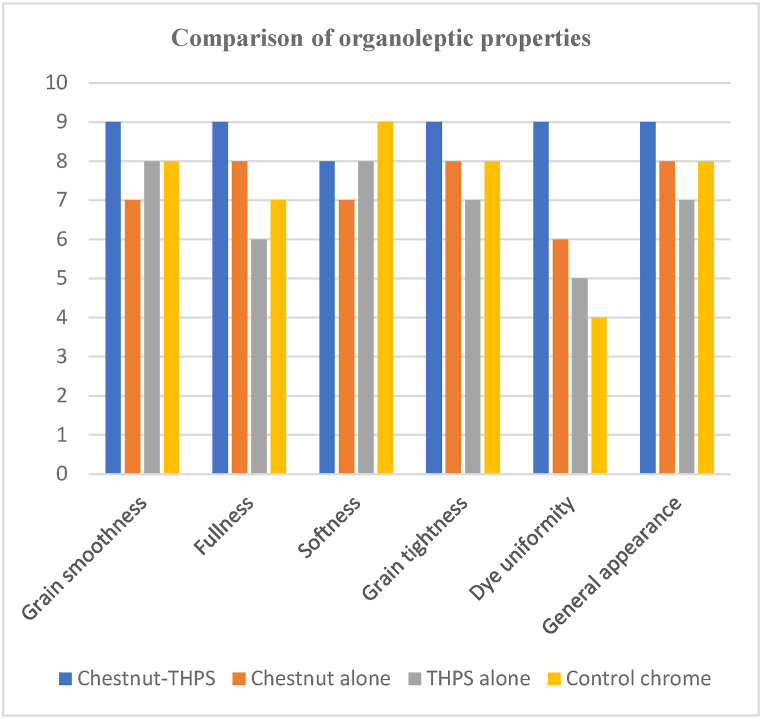
Graphical representation of organoleptic properties of crust leathers.

The bulk properties of the sheep upper leather, such as softness, grain smoothness, grain tightness, fullness, dye uniformity, and general appearance, both for the leathers produced using the combination and conventional tanning systems, were assessed by hand and visual inspection. The average values of the ratings for the leathers used were calculated for each functional attribute, and the findings are shown in Fig. 7. The assessment was done by inviting a group of experienced experts. Experts rated all the crust leathers from 0 to 10, with a reference to the best export-grade crust leather at 10. It was observed that crust leathers from experimental and control processes were found to have different organoleptic properties. The graph shows that the combination experimental crust leathers (chestnut-THPS) have better grain smoothness, fullness, grain tightness, and general appearance compared with leathers processed conventionally using chrome tanning system [36]. This is primarily due to the high tannin fixation and affinity of the experimental combination tannin materials for the pelt [65,66]. The problem of softness related to chestnut can be fixed by a proper mix of syntans along with the usage of penetrative synthetic fat liquors in post-tanned leather processing to impart the desired result [67]. The leathers tanned using the combination tanning system were observed to have a better general appearance than the other three tanning systems, and the chestnut-tanned leathers were seen to have the least general appearance. Similar findings of better softness, grain smoothness and general appearance were reported by Ref. [36] considering henna-THPS combination tanning system than henna alone. On the contrary, the enhancement in fullness, grain tightness and dye uniformity in this specific study while using the combination tanning system were reported by Ref. [36] to have no difference than using henna alone. The graphical representation of the organoleptic properties of leathers produced using all four tanning systems is shown in Fig. 7.

### 3.8 Chemical characteristics of crust leathers

Chemical characteristics analysis results such as percentages of fats and oils, moisture content, total ash content, and hide substance were determined both for the experimental and control leathers using standard procedures, and the results are presented in Table 11. The analysis was carried out for each sample in triplicate, and the results were tabulated in the form of the mean standard deviation. It was observed from the tabulated that the values of chemical characterization parameters such as fat and oil content, moisture content, and hide substance are all closer to each other for all the tanning systems. However, leather tanned using the conventional tanning system has the highest value of total ash content, which is an indication that leathers tanned conventionally using the chromium tanning system contain more non-volatile matter due to the presence of inorganic oxides and salt [[68], [69], [70]]. Leathers tanned utilizing combination tanning systems have chemical analysis results that are found to be within standard limits and quite typical, meeting the necessary standards for chemical analysis. Nevertheless, it was found that the percentage of total ash content in those leathers that had been traditionally tanned with chromium as a tanning agent was higher even above the recommended standard. The total ash content of the leathers tanned with the experimental tanning system was found to be nearly identical to that of the leathers reported by Ref. [36] utilizing a combination tanning system based on henna and THPS. Additionally, the results of the examination for the other parameters such as the percentage of fat and oil, the moisture content, and the percentage of hide substance were better than those that were published by Ref. [36].

**Table 11:** Chemical characteristics of experimental and control crust leathers.

Parameters	Leather samples				
	Chestnut-THPS (E)	Chestnut (alone)	THPS (alone)	Chrome (control)	Standard value
% Fat & oil content	6.7	8	7	8.6	15, Min
% Moisture content	14.9	13.4	14.2	14.4	–
% Total ash content	2.1	3.6	2.4	11.2	3–6
% Hide substance	70.9	68.6	69.1	71.5	64, Min

## 4 Conclusion

In the present study, an attempt has been made to use an eco-benign combination tanning system based on chestnut and THPS. Optimization of the combination tanning system based two tanning agents resulted in an optimum dosage of 20 % chestnut followed by 2 % THPS as tanning agents, providing the tanned leather with an optimum shrinkage temperature of 95 °C. The scanning electron microscopic analysis of the cross-section of the combination tanned leathers showed fiber coating due to the presence of THPS inside the leather matrix, leading to good structural stability and smoothness. The grain surface micrograph of the combination tanned leathers indicated the absence of surface deposition of tannins due to better tannin penetration, which is displayed on the crust leather as good fiber compaction and grain tightness properties. The filling nature of chestnut also gives us an admirable fullness. The organoleptic properties of the leathers processed with this combination tanning system are comparable to or better than those tanned using conventional chromium tanning systems. The physical strength characteristics of chestnut-THPS-tanned leathers are significantly enhanced due to the presence of THPS. This could be due to a higher level of crosslinking between the skin matrix fiber structure and the tannins utilized in the combination tanning system. As they are the only parameters that distinguish natural leather from synthetics, hygiene properties are the most necessary parameters for any type of leather. Accordingly, the test results in this study revealed the chestnut-THPS combination tanned leathers to have better water vapor permeability and percentage of water vapor absorption than the conventional chrome and chestnut tanned leathers. The chemical characterization demonstrated similarity in the analysis results for all the tanning systems except for the leather tanned using THPS alone. In general, in the combination tanning system considering chestnut and THPS, it was observed that the leather had better physical strength characteristics and organoleptic properties; furthermore, this tanning system was found to be important in the reduction of effluent load by lowering the TS, TDS, TSS, and BOD of the spent liquor. Finally, it can be concluded that the leathers tanned using the chestnut-THPS combination tanning system can meet the mandatory and voluntary conditions specified by environmental protection organizations in terms of allowable waste water discharge limits.

## Ethical approval and consent to participate

All procedures are carried out in accordance with the institution's policies and rules. The institution's ethical clearance committee gave its approval to each experimental protocol.

## Funding

Not applicable. Only facilities are provided.

## Consent for publication

Not applicable.

## Data availability

All data sets generated during this study are included in this published article.

## CRediT authorship contribution statement

**Haftom Girmay:** Writing - review & editing, Writing - original draft, Software, Resources, Methodology, Investigation, Formal analysis, Data curation, Conceptualization. **Ashagrie Mengistu:** Writing - review & editing, Writing - original draft, Software, Methodology, Formal analysis, Data curation. **Berhanu Assefa:** Writing - review & editing, Writing - original draft, Visualization, Supervision, Methodology, Formal analysis, Conceptualization. **Mikiyas Abewaa:** Writing - review & editing, Writing - original draft, Software, Methodology. **Getaneh Andualem:** Writing - review & editing, Writing - original draft, Software, Resources. **Bereket Yeheyis:** Writing - review & editing, Software, Methodology.

## Declaration of competing interest

The authors declare that they have no known competing financial interests or personal relationships that could have appeared to influence the work reported in this paper.
