# Supplementary material for: The development of chrome free chestnut and Tetrakis Hydroxymethyl Phosphonium Sulfate based Eco-benign combination tanning system
Source: Heliyon. 2023 Dec 6;10(1):e23141. doi: 10.1016/j.heliyon.2023.e23141 (PMC10756982; doi:10.1016/j.heliyon.2023.e23141)
Supplement: Multimedia component 1 [file mmc1.pdf]

<u>Form to confirm authorship changes for *Heliyon*</u>

This form must be ***signed by all authors*** when there is a change in authorship which includes changes to any of the following items: authorname(s), order of the authors, the corresponding author(s), the addition of authors, the removal of authors and changes in affiliation.

By personally signing this note, **all** authors confirm that: I) the changes are in accordance with their scientific contribution, II) they agree with allthe changes and III) confirm that the authorship list conforms to the authorship criteria outlined on *Heliyon*'s ethics page. IV) it is the responsibility of the corresponding author to get the signature from all co-authors accepting the change. In case of any ethic violation/malpractice in the signature, the corresponding author is accountable. The completed form should be returned along with the final/revised manuscript to proceed further with the manuscript. Manuscripts for which incomplete forms have been submitted will be rejectedwithin 5 working days.

Any disputes on the authorship list and contributions need to be resolved by the involved scientists and *Heliyon* will only proceed with the evaluation of the manuscript once we receive confirmation, through this form, that such an agreement between the authors has been reached.

Heliyon **will not accept changes to the authorship list in the late stages of the editorial process (when a paper is in Accept in Principle stage, acceptance or after publication)**

Manuscript number:

HELIYON-D-23-42639

Article title:

The development of chrome free chestnut and Tetrakis Hydroxymethyl Phosphonium Sulfate based Eco-benign combination tanning system.

Complete new author list:

1. Haftom Girmay Gebru
2. Ashagrie Mengistu Kebede
3. Berhanu Assefa Demissie
4. Mikiya Abewaa Gnaro
5. Getaneh Andualem Eyasu
6. Bereket Yeheyis belay

Date: 06-Nov-2023

#	First name	Last name	Dept. & Institutionname	Institutional email address	Order change(Y/N)	Addition / Deletion	Change in Authorname (Y/N)	Affiliation Change (Y/N)	Reason for the change	Signature
1	Haftom	Gebru	Research and Technology Development, Manufacturing Industry Development Institute and Addis Ababa Institute of Technology	buluqsaafuttaasaa@gmail.com	Yes, but only in the system. The order in the manuscript is still the same as the originally submitted manuscript.	No	No	No	The order has not been changed with respect to the originally sent manuscript. But we just tried to solve the mistake happen during sending of the manuscript that the system automatically selected the corresponding author as a first author. However, the reality was that Mr. Haftom is the first contributor of the research work. It is only due to technical problem that first author was changed while uploading in the system. In the original manuscript Mr. Haftom was affiliated as a first author.	
2	Ashagrie	Kebede	Research and Technology Development, Manufacturing Industry Development Institute	Ashagmen2017@gmail.com	Yes, to make the order in the system the same as the order in the original manuscript.	No	No	No	Because Mr. Haftom is the first contributor of the research work and in the original manuscript, he was first author. The problem happened when listing authors in the system	
3	Birhanu	Demissie	Addis Ababa Institute of Technology	obsinaanabawa@gmail.com	Yes, but only in the system to make it the same as the order in the originally submitted manuscript	No	No	No	Because Mr. Haftom was the first contributor in the original manuscript too.	
4	Mikiyas	Gnaro	Department of Chemical Engineering, College of Engineering and Technology, Wachemo University	mikiyasabewaa02@gmail.com	Yes, but Only in the system and the order in the manuscript still takes the order the originally submitted manuscript.	No	No	No	It is to correct the technical problem that happened while listing authors in the system.	

5	Getaneh	Eyasu	Manufacturing Industry Development Institute, leather and Leather products Industry Research and Development Center	abebeoro481@gmail.com	Yes, but only in the system to make it similar with the order in the manuscript.	No	No	No	Because in the original manuscript Mr. Haftom is the first author and he was the first contributor of the research.	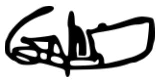
6	Bereket	Belay		abewaamikiyas@gmail.com	Yes, but Only in the system. The order in the manuscript has no problem.	No	No	No	Just to correct technical problem happened while sending the manuscript.	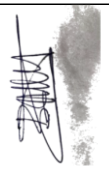

Dear Wen Xia,

We are not trying to change order, as you can see in the original manuscript Mr. Haftom was affiliated as a first author. Unfortunately, during sending the manuscript may be the system automatically considered the corresponding author as the first author by mistake. We also observed it in the generated PDF of the manuscript, that is why we tried to make the order in the system similar with that in the originally sent manuscript. Would you please consider it a technical problem.

The other thing that we want to make you know is that we used our gmail account in the place of Institutional e-mail address because we don’t have an institutional email address.
